# Supplementary material for: Haldane topological spin-1 chains in a planar metal-organic framework
Source: Nat Commun. 2023 Sep 6;14:5454. doi: 10.1038/s41467-023-41014-1 (PMC10482874; doi:10.1038/s41467-023-41014-1)
Supplement: Supplementary file 1 — Supplementary information [file 41467_2023_41014_MOESM1_ESM.pdf]

Supplementary Information

Haldane spin-1 chains in a planar metal-organic framework

Tin et al.

Supplementary Methods. *Synthesis of NiBO and NiBO-d_8 and characterization by powder X-ray diffraction and IR spectrum*……………….…..……..S-2

Supplementary Note 1. *Crystal structure of NiBO-d_8*……..…………………………….....S-6

Supplementary Note 2. *Additional powder neutron diffraction (PND) data for NiBO*……..S-12

Supplementary Note 3. *Additional magnetic susceptibility (DC) data*……….……………...S-13

Supplementary Note 4. *Additional HFESR results*…………………..…………………….S-15

Supplementary Note 5. *Additional Quantum Monte Carlo (QMC) results*……………….....S-16

Supplementary Note 6. *Additional specific-heat data*……………………………….……S-26

Supplementary Note 7. *Additional INS spectra, tables of phonon symmetries, and spin densities*…………………………………………………...…...S-27

CIF (Crystallographic Information Framework) file for the crystal structure of NiBO-d_8 is given in the Source Data.

Supplementary Methods

Synthesis of NiBO and NiBO-d_8 and characterization by powder X-ray diffraction and IR spectrum

The chemicals were purchased commercially and used without further purification, including the following: nickel(II) bromide trihydrate [$NiBr_2$•$3H_2O$, 98%, Alfa Aesar], oxalic acid dihydrate [$(COOH)_2$•$2H_2O$, 99.5+%, Acros Organics], 4,4'-bipyridine [$C_{10}H_8N_2$, 98%, Acros Organics], 4,4'-bipyridine-d_8 [$C_{10}D_8N_2$, 98%-d_8, CDN Isotopes].

For the discussion below, we will address the protonated version of [Ni(μ-4,4′-bpy)(μ-ox)]$_n$ as NiBO and the deuterated version as NiBO-d_8.

Polycrystalline samples of NiBO were prepared as reported in the literature.[1] Repeated attempts at synthesizing NiBO were unsuccessful in obtaining single-crystals larger enough for a single-crystal X-ray diffraction measurement but attempts at synthesizing NiBO-d_8 yielded single-crystals for X-ray diffraction measurements, as discussed below. The powder X-ray diffraction (PXRD) patterns of NiBO compared to the reported calculated PXRD pattern from the reported structure[1] can be seen in Supplementary Figure 1. The PXRD measurements for NiBO were taken at room temperature, while the reported calculated PXRD patterns were obtained from the reported single-crystal X-ray diffraction data at 295 K. The two PXRD patterns are similar up to 50 degrees. Since we did not obtain single crystals of NiBO, we are unable to compare the unit cells to the reported structure. On the other hand, as we were successful at obtaining single crystals of NiBO-d_8, we collected the single-crystal X-ray diffraction data and discussed them below, including a comparison to the reported structure as seen in Supplementary Table 1.

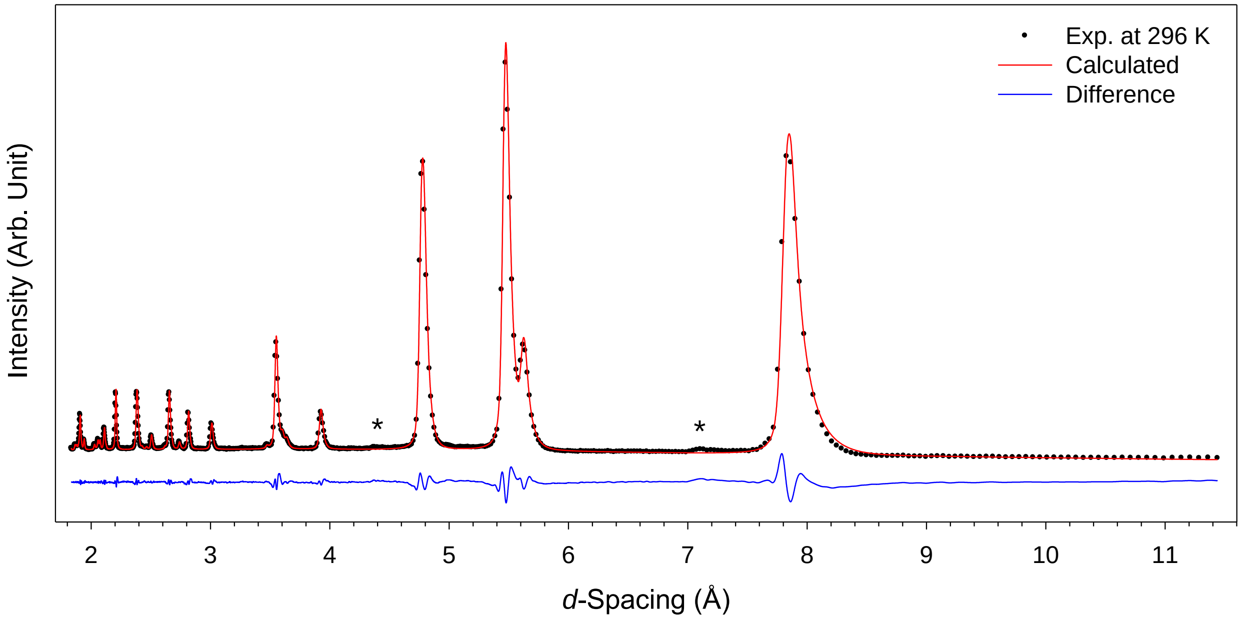

Supplementary Figure 1. Experimental PXRD pattern of NiBO compared to the PXRD pattern calculated by the Pawley method. *: impurity peak. Source data are provided as a Source Data file.

As indicated above, we could only obtain polycrystalline samples from the synthesis of NiBO. On the other hand, single-crystal and polycrystalline samples of NiBO-d_8 were prepared through hydrothermal synthesis described below.

Nickel(II) bromide trihydrate [$NiBr_2$•$3H_2O$, 0.2731 g, 1.002 mmol], oxalic acid dihydrate [$(COOH)_2$•$2H_2O$), 0.1263 g, 1.002 mmol], and 4,4′-bipyridine-d_8 [$C_{10}D_8N_2$, 0.2469 g, 1.503 mmol] were mixed in 8 mL of deionized (DI) H_2O inside a 25 mL Teflon lined autoclave. The mixture was stirred for 2 minutes. The autoclave was then sealed and was placed into a furnace with proper insulation at 168 °C for 7 days. Afterwards, the furnace was turned off and the autoclave was naturally cool down to room temperature inside the insulated furnace over 24 hours. The cool down process to room temperature only took about 10 hours, but the reaction vessel was left undisturbed for up to 24 hours afterwards to let the single crystals grow bigger in

size. After 24 hours, the mixture was filtered and washed with acetone and DI H_2O. The solid sample contains a mixture of single-crystal and polycrystalline forms. The filtrate has a dark blue color. The polycrystalline sample of NiBO-d_8 MOF has a light blue color, and the single-crystals of NiBO-d_8 MOF are blue plate-shaped crystals large enough for a single-crystal X-ray diffraction measurement. The diffraction data for NiBO-d_8 are shown in Supplementary Table 1 below. The total yield is 0.2014 g or 66.4% based on the $NiBr_2$•$3H_2O$ and $(COOH)_2$•$2H_2O$. Supplementary Figure 2 shows a comparison between the experimental PXRD patterns of polycrystalline NiBO-d_8 at room temperature and the calculated PXRD patterns from the single-crystal structure of NiBO-d_8 at 100(2) K. The PXRD patterns are similar. Supplementary Figure 3 shows the IR spectrum of the polycrystalline NiBO-d_8.

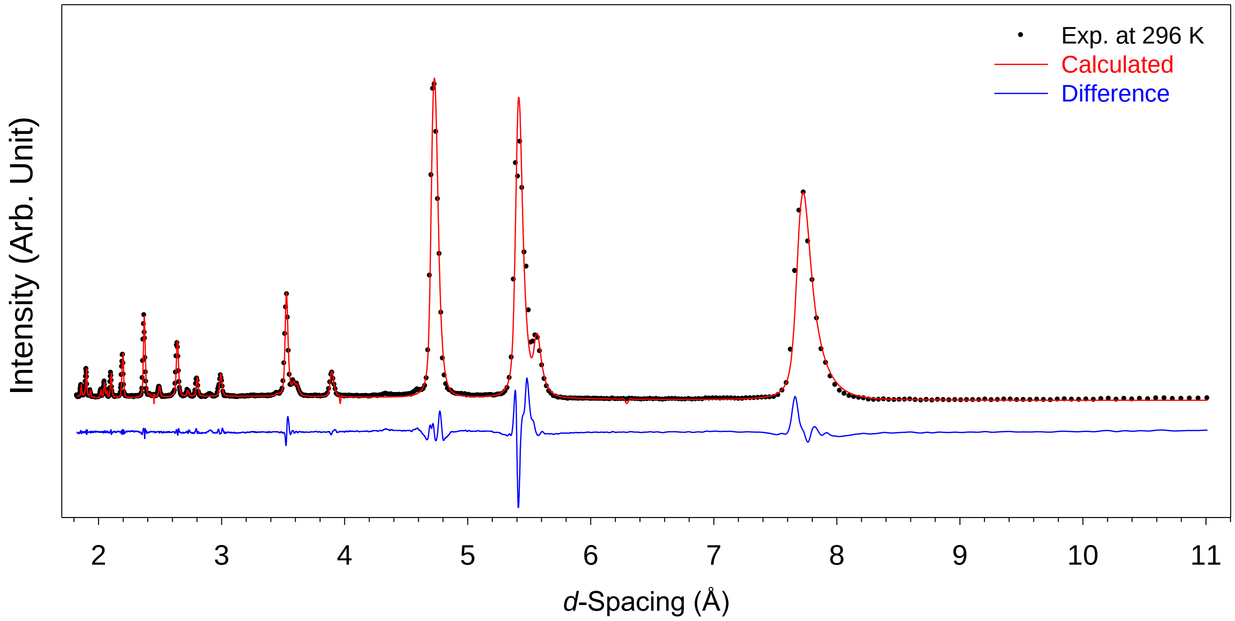

Supplementary Figure 2. Experimental PXRD patterns of NiBO-d_8 at 296 K compared to the PXRD pattern calculated by the Pawley method. Source data are provided as a Source Data file.

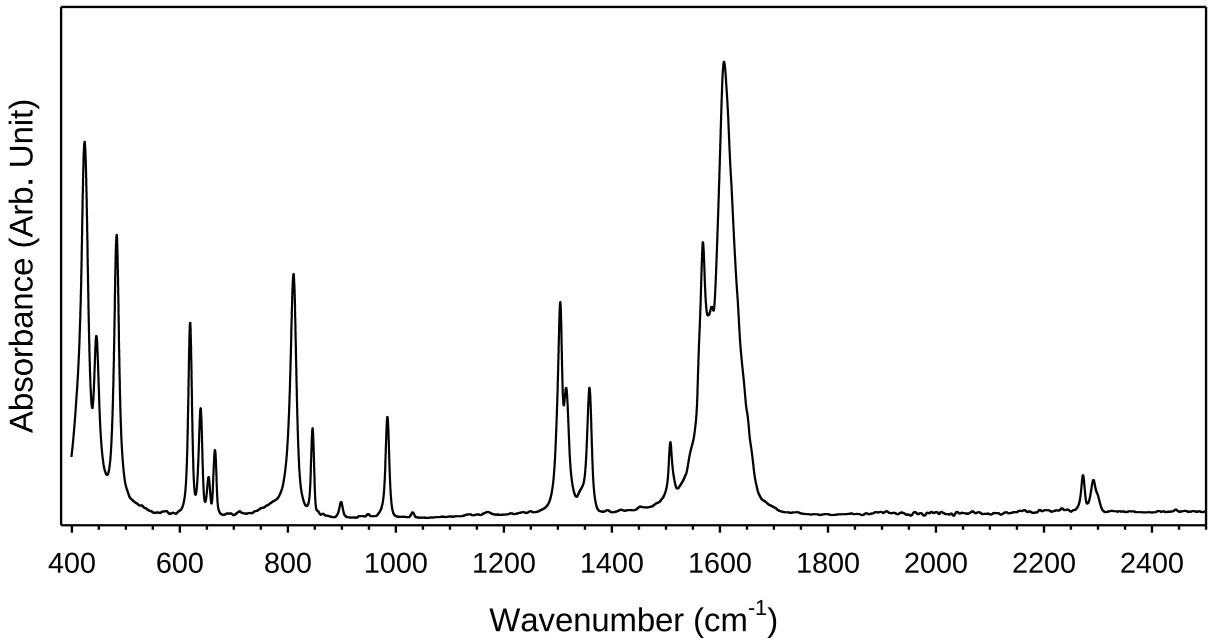

Supplementary Figure 3. The IR spectrum of NiBO-d_8 from 400 to 2500 cm^{-1} at room temperature. Source data are provided as a Source Data file.

Supplementary Note 1. *Crystal structure of NiBO-d_8*

Supplementary Table 1 includes crystal information from the current studies measurements at 100(2) K and the reported 295 K measurements.[1] The single-crystal X-ray diffraction data for NiBO-d_8 were solved using hydrogen instead of deuterium due to the two elements having indistinguishable pattern in the X-ray diffraction measurements. The reported crystal structures at 295 K and NiBO-d_8 at 100(2) K are mostly similar with some major differences, such as solving the structure of NiBO-d_8 in the monoclinic *C*2 (No. 5) space group instead of the orthorhombic *Immm* (No. 71). Continuous shape measurement (CShM) was performed on the crystal structure at 100 K. The shape of the structure is 19.860 deviating from the perfect octahedral shape at 0. The local structure shows a Ni^{2+} ion coordinates with two oxalate ligands in the *x*-*y* plane, while two 4,4′-bipyridine ligands coordinates in the *z*-direction. Supplementary Figure 4 shows the local structure around the Ni^{2+} ion. The Ni^{2+} ions across the oxalate ligands form the Haldane chains, while the 4,4′-bipyridine ligands connect the individual chains to form the 2D framework. Some of the selected bond lengths and bond angles around the Ni^{2+} ion at 100 K are in Supplementary Table 2. The Ni1 to N1 and N2 bond lengths are a bit longer than that of the Ni1 to O1 and O2 bond lengths. The bond angles of O1-Ni1-O1 and O2-Ni1-O2 are 179.4(2) and 179.5(2), respectively. The angles suggest that the two oxalate ligands are in the same plane (*x*-*y* plane) as one another. While the bond angles of O2-Ni1-N1 and O2-Ni1-N2 are very close to 90 degrees indicating that the two 4,4′-bipyridine ligands are in the axial positions. Fig. 3 and Supplementary Figure 5 show the inter- and intra-chain direction of the Haldane 1D chain MOF as well as the view down the *a*-axis. The view of the crystal structure down the *b*- and *c*-axis can be seen in Supplementary Figures 6 and 7.

At 100 K, the refinement of the crystal structure placed the two pyridine rings in the 4,4′-

bipyridine ligands rotated at two different planes, while the reported structure (295 K) shows the bipyridine rings to be in the same plane. The freezing of the bipyridine rings rotation places the structure at 100 K into a lower symmetry than that of the structure at 295 K. The rotation of the two pyridine rings allows a better understanding of the magnetic coupling strength between the two nearest nickel atoms along the inter-chain direction. With the two pyridine rings rotated off the plane to one another, the magnetic coupling strength (*J*) in the inter-chain direction is expected to be negligible. The result is favorable for the Haldane phase to exist in the form of a 2D-MOF. Therefore, the nickel chains that form across the oxalate ligands can be effectively considered as individual isolated Haldane 1D chains in the system.

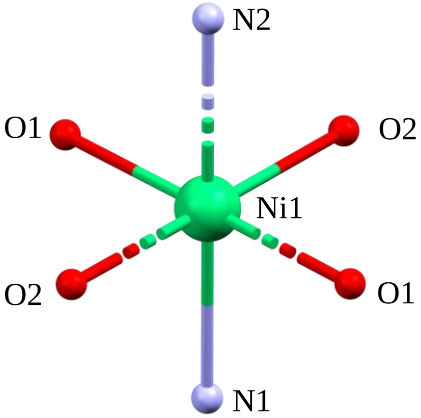

Supplementary Figure 4. The local structure around the Ni^{2+} ion in NiBO-d_8. Green: Ni; Red: O; Purple: N; Gray: C. The CIF file for the crystal structure is provided as a Source Data file.

Supplementary Table 1. Comparison of crystallographic data for [Ni(μ-4,4′-bpy-d_8)(μ-ox)]$_n$ (NiBO-d_8) at 100 K (current studies) and [Ni(μ-4,4′-bpy)(μ-ox)]$_n$ (NiBO) at 295 K (reported)

	**100(2) K**	**295 K**[1]
Empirical formula	$C_{12}D_8N_2NiO_4$	$C_{12}H_8N_2NiO_4$
Fw	310.95	N/A
Temp (K)	100(2)	295
Crystal system	Monoclinic	Orthorhombic
Space group	*C*2 (No. 5)	*Immm* (No. 71)
a, Å	12.096(2)	5.3030(11)
b, Å	11.288(2)	10.955(2)
c, Å	5.3238(10)	11.257(2)
α, °	90	90
β, °	115.995(5)	90
γ, °	90	90
V, Å^3	653.4(2)	653.968
Z	2	2
D_{calc}, g/cm^3	1.540	1.538
Crystal size (mm^3)	0.062 × 0.220 × 0.258	N/A
θ range (deg)	2.601–30.993	N/A
Reflections collected	2079	N/A
Independent reflections	13697 [R(int) = 0.0297]	N/A
Completeness to θ	100%, 30.993°	N/A
GOF on F^2	0.816	N/A
Final R indices	$R1 = 0.0160$	$R1 = 0.024$
$[I > 2\sigma(I)]^a$	w$R2 = 0.0394$	w$R2 = 0.024$
R indices (all data)	$R1 = 0.0161$	N/A
	w$R2 = 0.0394$	N/A
Largest diff. peak and hole, e.Å^{-3}	0.463 and −0.390	N/A

^{a} $R = \Sigma ||F_o| - |F_c|| / \Sigma|F_o|$; $R_w = (\Sigma[w(F_o^2 - F_c^2)^2] / \Sigma[w(F_o^2)^2])^{1/2}$

Supplementary Table 2. Selected bond lengths and bond angles around the Ni^{2+} ion in NiBO-d_8 at 100 K

Bond Lengths (Å)	
Ni1-N1	2.092(5)
Ni1-N2	2.092(5)
Ni1-O1	2.0488(10)
Ni1-O1	2.0489(10)
Ni1-O2	2.0533(9)
Ni1-O2	2.0533(9)
**Bond Angles (°)**	
O1-Ni1-O1	179.4(2)
O1-Ni1-O2	97.47(4)
O1-Ni1-O2	82.53(4)
O1-Ni1-O2	82.53(4)
O1-Ni1-O2	97.47(4)
O2-Ni1-O2	179.5(2)
O1-Ni1-N1	90.32(11)
O1-Ni1-N1	90.32(11)
O2-Ni1-N1	89.73(11)
O2-Ni1-N2	89.73(11)
O1-Ni1-N2	89.68(11)
O1-Ni1-N2	89.68(11)
O2-Ni1-N2	90.27(11)
O2-Ni1-N2	90.27(11)
N1-Ni1-N2	180.0

(a)

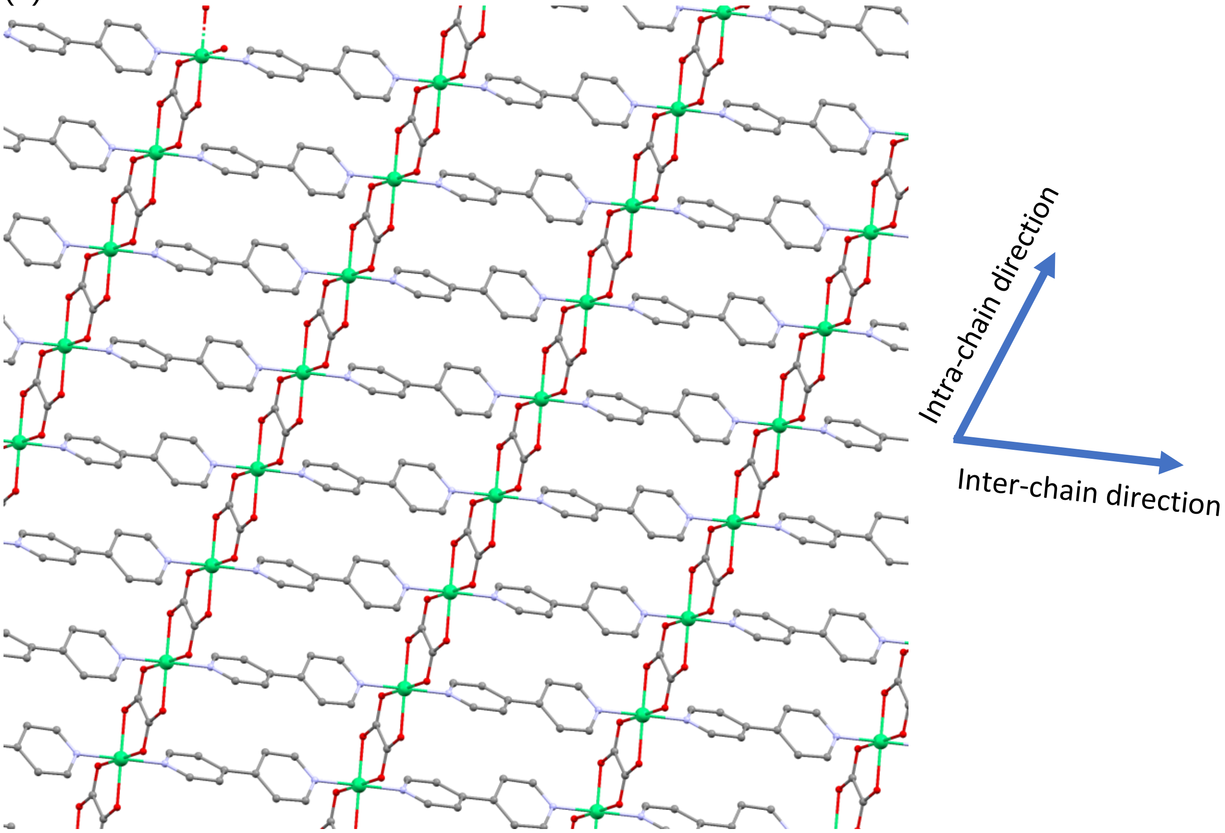

(b)

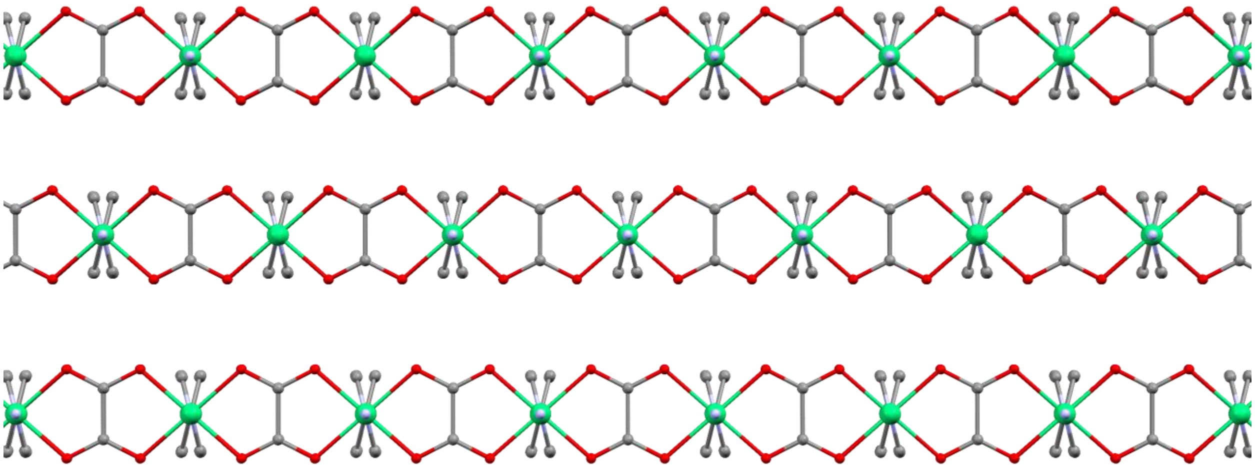

Supplementary Figure 5. (a) Crystal structure of NiBO-d_8 showing inter-chain and intra-chain directions. (b) Crystal structure of NiBO-d_8 viewed down the crystallographic *b*-axis. Green: Ni; Red: O; Purple: N; Gray: C. D atoms are omitted for clarity.

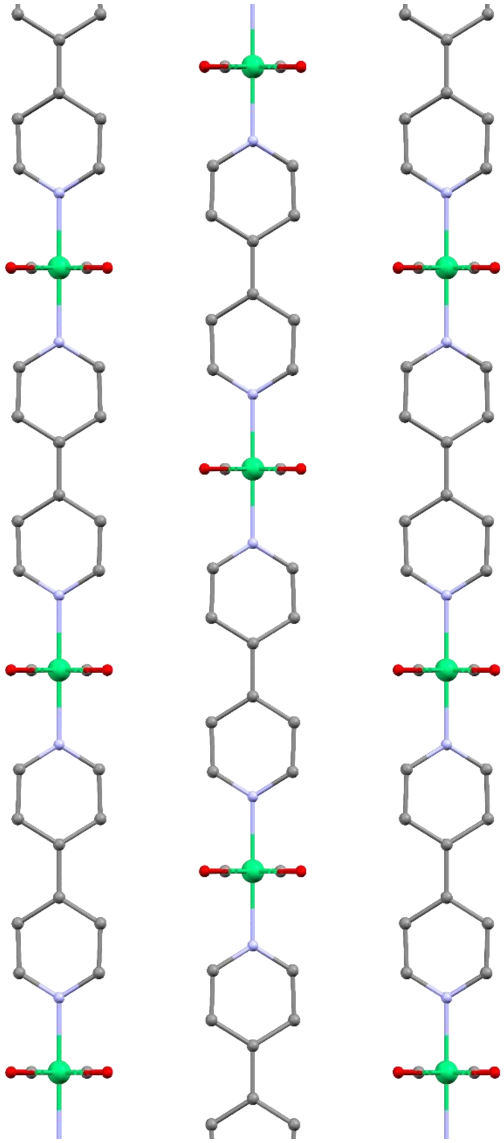

Supplementary Figure 6. Crystal structure of NiBO-d_8 view down the crystallographic *c*-axis.

Green: Ni; Red: O; Purple: N; Gray: C. D atoms are omitted for clarity.

Supplementary Note 2. *Additional powder neutron diffraction (PND) data for NiBO*

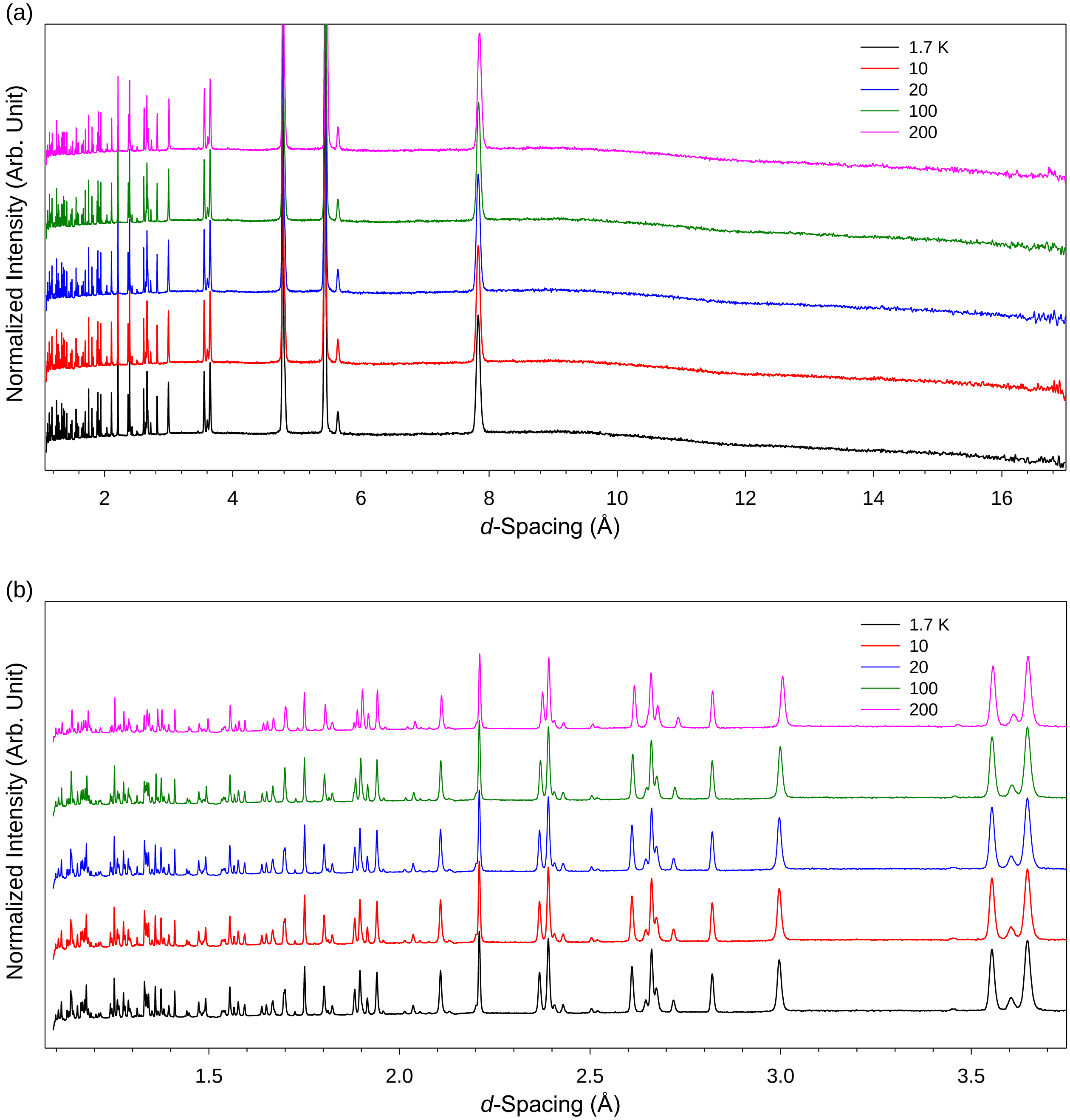

Supplementary Figure 7. PND data for NiBO at 1.7 K, 10 K, 20 K, 100 K and 200 K. The data at 1.7 K and 200 K are given in Fig. 4a and are reproduced here for comparison. (a) All *d*-spacings. (b) Expansion of the low *d*-spacing region. Source data are provided as a Source Data file.

Supplementary Note 3. *Additional magnetic susceptibility (DC) data*

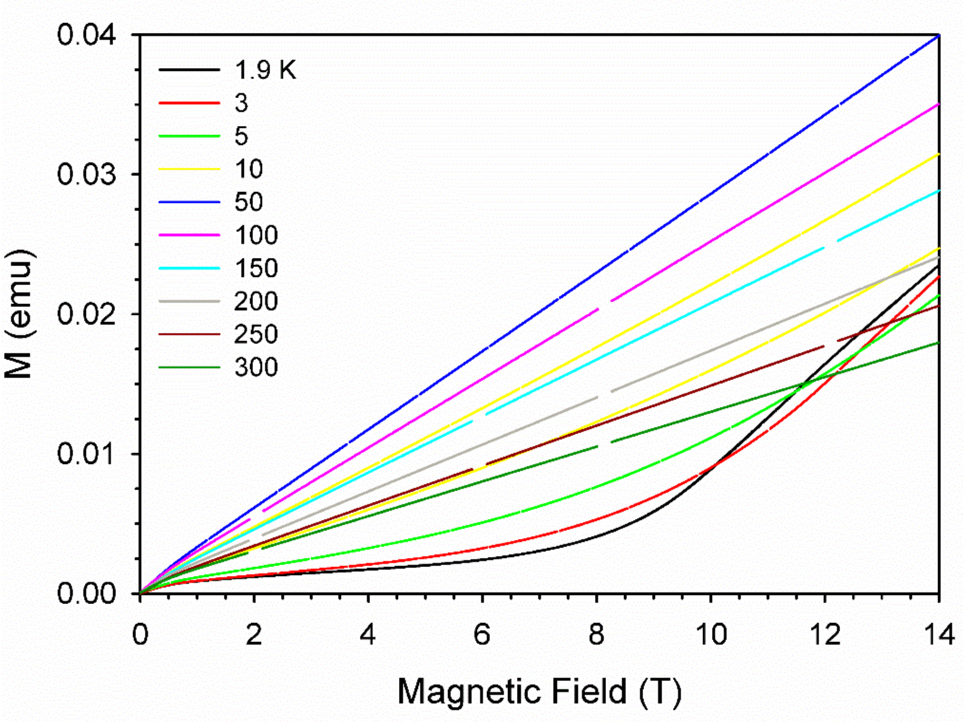

Supplementary Figure 8. Variable-field DC magnetization of NiBO at various temperatures. Source data are provided as a Source Data file.

Background subtraction of the DC magnetic susceptibility data were performed using the equation: $\chi = C/(T - \theta_{CW}) + \chi_{para}$, where χ_{para} is the paramagnetic contribution in the background.[2] Supplementary Figure 9a shows the data after background subtraction and Supplementary Figure 9b before background subtraction for each of the magnetic field. χ_{para} is constant and temperature-independent. The χ_{para} value is not only consisting of paramagnetic or diamagnetic contributions in the system but also the background noise of the instrument as well. Supplementary Figure 10 shows the fit to find χ_{para} between 200 and 300 K for 0.1 T data. The same fitting process was performed for the data at other magnetic field strength.

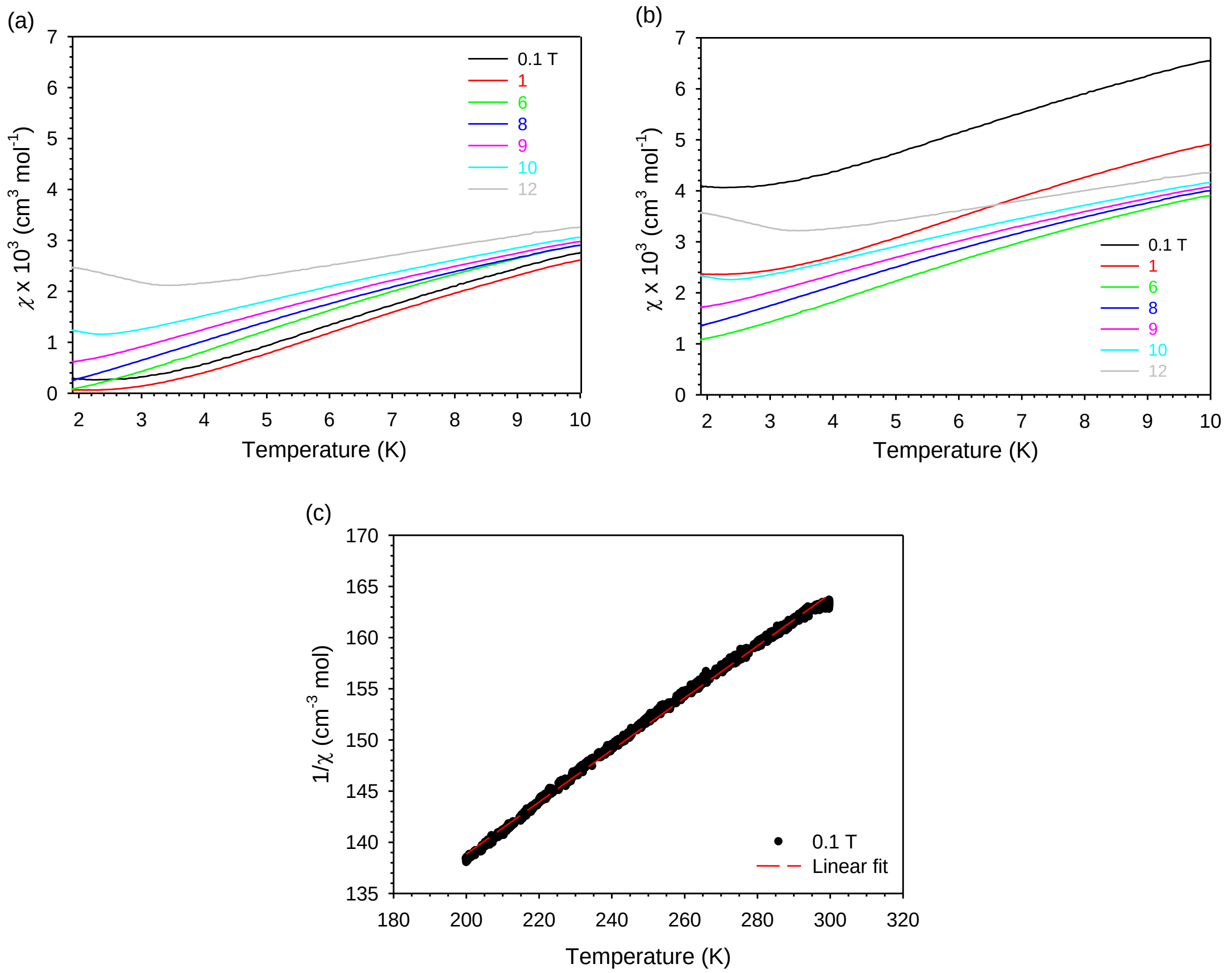

Supplementary Figure 9. (a-b) Comparison between DC magnetic susceptibility data of NiBO: (a) After background subtraction using χ_{para}. This is Fig. 5b reproduced here for comparison. (b) Before background subtraction using χ_{para}. (c) Fitting of DC magnetic susceptibility data of NiBO between 200 and 300 K at 0.1 T. The best fit value gives χ_{para} = 0.0033 cm^3 mol^{-1}. Source data are provided as a Source Data file.

Supplementary Note 4. *Additional HFESR results*

Frequency dependence of the resonance observed by HFESR is given in Supplementary Figure 10 and discussed in the main text. Comparison of the frequency dependence of the resonance by HFESR with magnetic field strength vs. difference $\Delta_3 - \Delta_1$ from QMC simulations is given in Supplementary Figure 17.

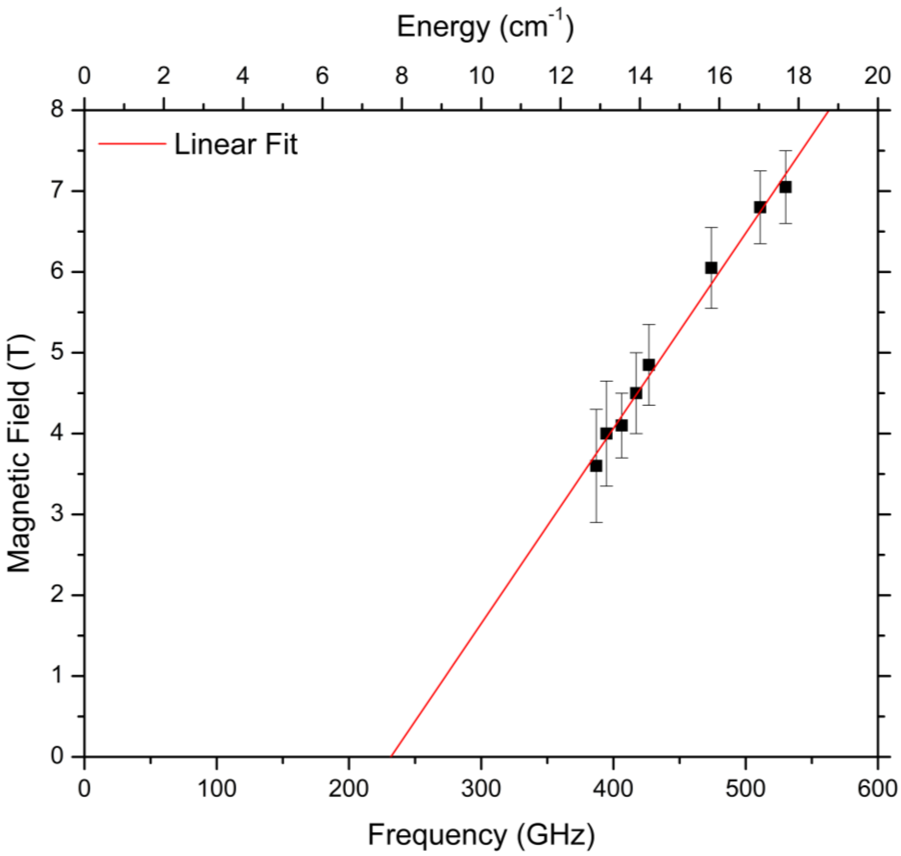

Supplementary Figure 10. Frequency dependence of the resonance in NiBO observed by HFESR. The red line is a linear fit to the data. Error bars of the magnetic fields are shown. Source data are provided as a Source Data file.

Supplementary Note 5. *Additional Quantum Monte Carlo (QMC) results*

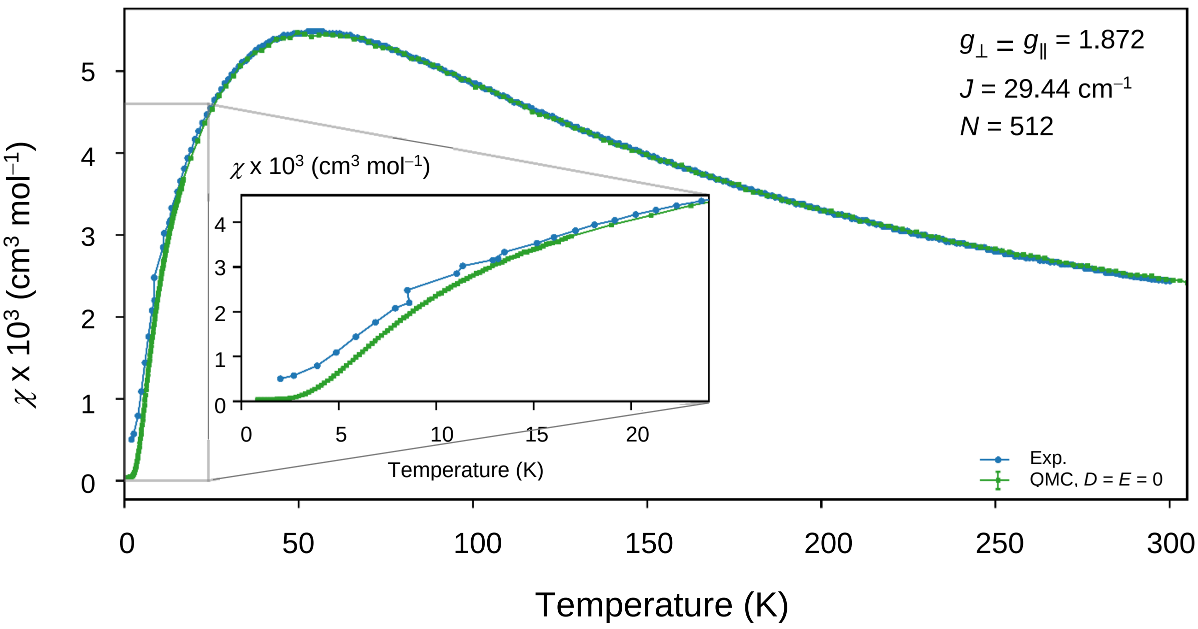

Supplementary Figure 11. Magnetic susceptibility of NiBO vs temperature. QMC simulations are carried out for N = 512 sites and without any additional anisotropies. Comparing the simulations to the experimental data gives J = 29.44 cm^{-1}, g = 1.872, and $\chi_{para} \approx 10^{-5}\ cm^3\ mol^{-1}$. Source data are provided as a Source Data file.

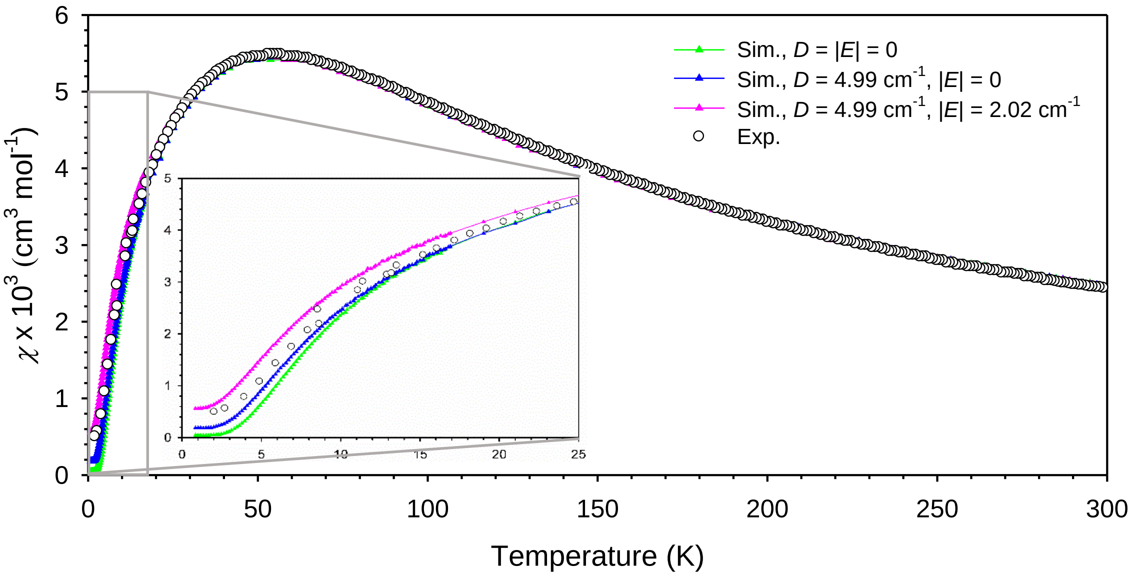

Supplementary Figure 12. Magnetic susceptibility of NiBO vs temperature. QMC simulations are carried out for N = 512 sites and with a D and E term. We further show results without any anisotropies. For J, $g_{\perp} = g_{\parallel}$ and χ_{para} we used the same values (with and without anisotropies). Source data are provided as a Source Data file.

Before analyzing the low temperature behavior of the magnetic susceptibility, we note that due to the transverse contribution in the powder averaging the susceptibility goes to a finite value in the zero-temperature limit. Therefore, we expect the following (asymptotic) low temperature behavior:

$$\chi = \chi(T = 0) + \alpha e^{-\Delta_1/T}$$

where α denotes some constant and Δ_1 is the lowest spin gap. Therefore, we expect on a log-scale, a linear behavior:

$$\log\big(\chi - \chi(T = 0)\big) = -\frac{\Delta_1}{T} + \text{const.}$$

The zero-temperature limit $\chi(T=0)$ can be readily extracted from the QMC simulations. In Supplementary Figure 13, the spin gap (Δ_1) is in good agreement with the experimental susceptibility data, excluding the lowest two data points. The slight enhancement of the experimental magnetic susceptibility at the lowest two temperatures is probably due to a very weak paramagnetic contamination.

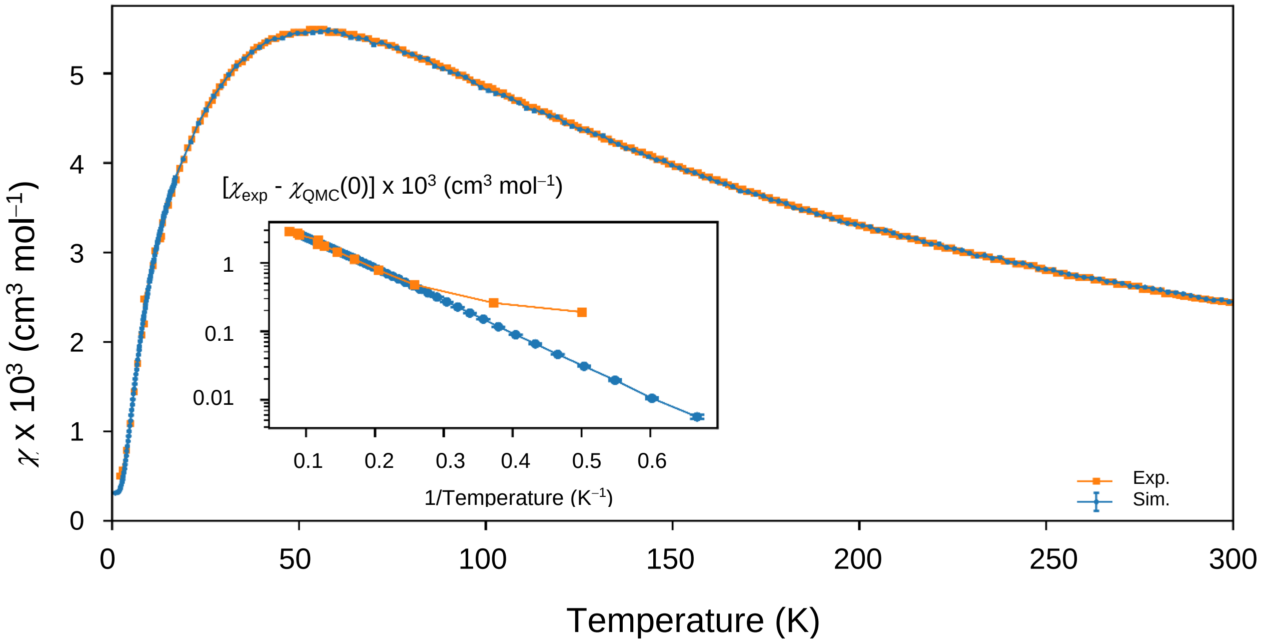

Supplementary Figure 13. Magnetic susceptibility of NiBO as a function of temperature as obtained from experiments and QMC simulations. Error bars in the QMC simulations of the magnetic susceptibility are shown. The inset shows the susceptibility subtracted by the zero-temperature limit on a logarithmic scale as a function of inverse temperature. Source data are provided as a Source Data file.

Regarding the splitting of the triplet state, one obtains in first-order perturbation theory the gaps as given in Eq. 5 of the main text.[3,4] If an additional magnetic field (H') is applied to the system, the triplet states shift further. One can consider in the canonical basis an additional

Zeeman term, which leads to a perturbation of the form:[3]

$$H_s + \mu_B g H' \cdot S = \begin{pmatrix} \Delta_x & -i\mu_B g {H'}_z & i\mu_B g {H'}_y \\ i\mu_B g {H'}_z & \Delta_y & -i\mu_B g {H'}_x \\ -i\mu_B g {H'}_y & i\mu_B g {H'}_x & \Delta_z \end{pmatrix}$$

where *H'* denotes the magnetic field in Tesla. From diagonalizing the effective Hamiltonian above, one obtains the field dependence of the triplet states for an arbitrary direction of the field, as shown in Supplementary Figures 14 and 15.

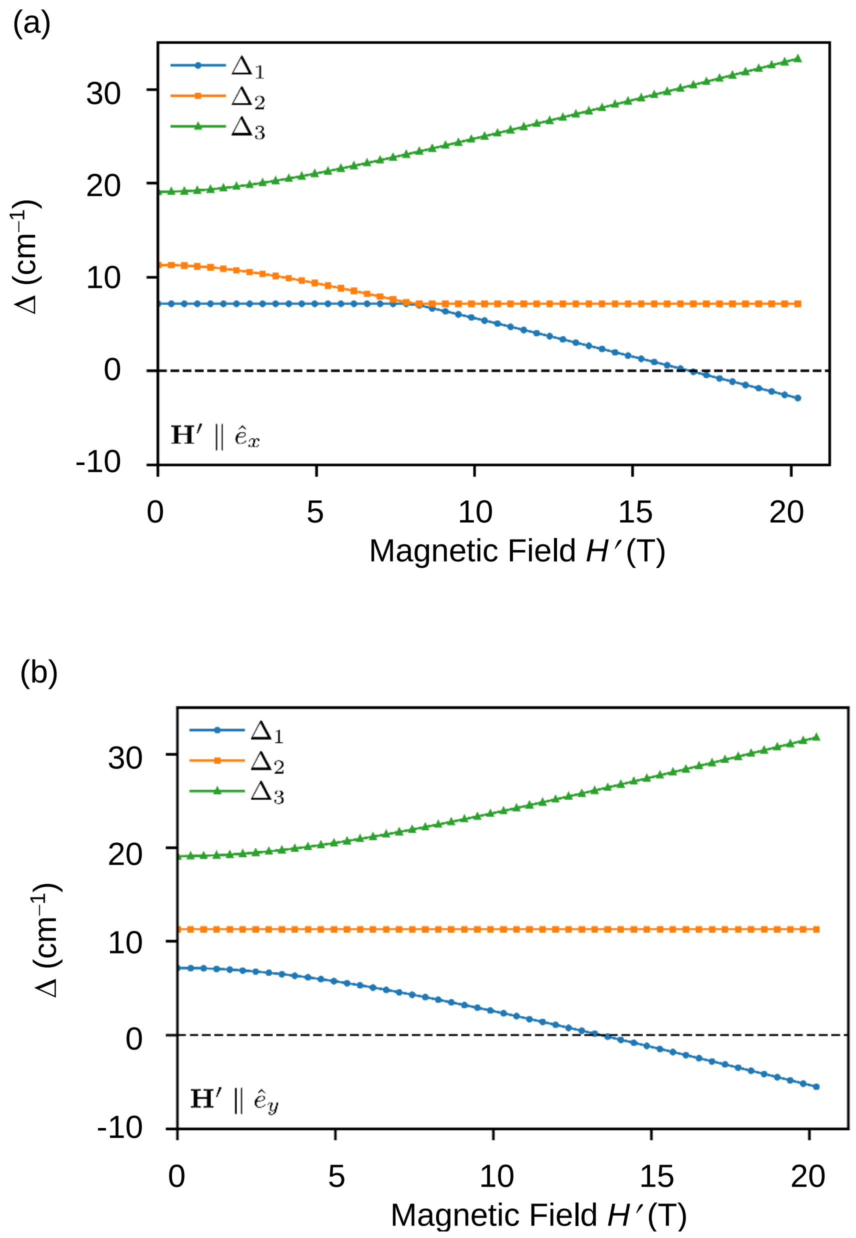

Supplementary Figure 14. Eigenvalues Δ_1, Δ_2, Δ_3 as functions of the magnetic field strength ($H' = |\boldsymbol{H}'|$). (a) $\boldsymbol{H}' \parallel \hat{e}_x$. $\hat{e}_x$ is the unit vector (length = 1) along the *x*-axis. (b) $\boldsymbol{H}' \parallel \hat{e}_y$. $\hat{e}_y$ is the unit vector (length = 1) along the *y*-axis. Source data are provided as a Source Data file.

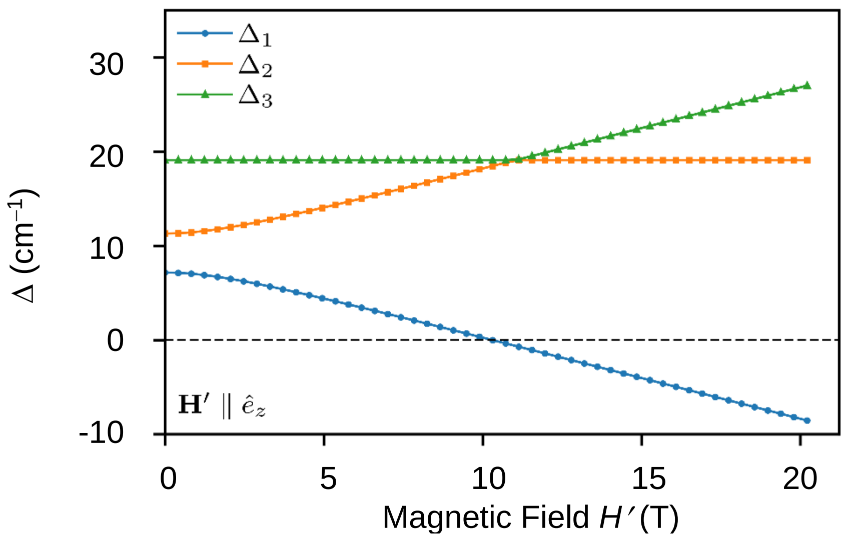

Supplementary Figure 15. Eigenvalues Δ_1, Δ_2, Δ_3 as functions of the magnetic field strength for $\boldsymbol{H}' \parallel \hat{e}_z$. $\hat{e}_z$ is the unit vector (length = 1) along the *z*-axis. Source data are provided as a Source Data file.

For a powder sample, we must average over all field directions. Therefore, we drew 1000 random samples at each field strength to sample different directions yielding the gaps shown in Supplementary Figure 16. From the varying field directions, we obtain broad distributions for larger field strengths.

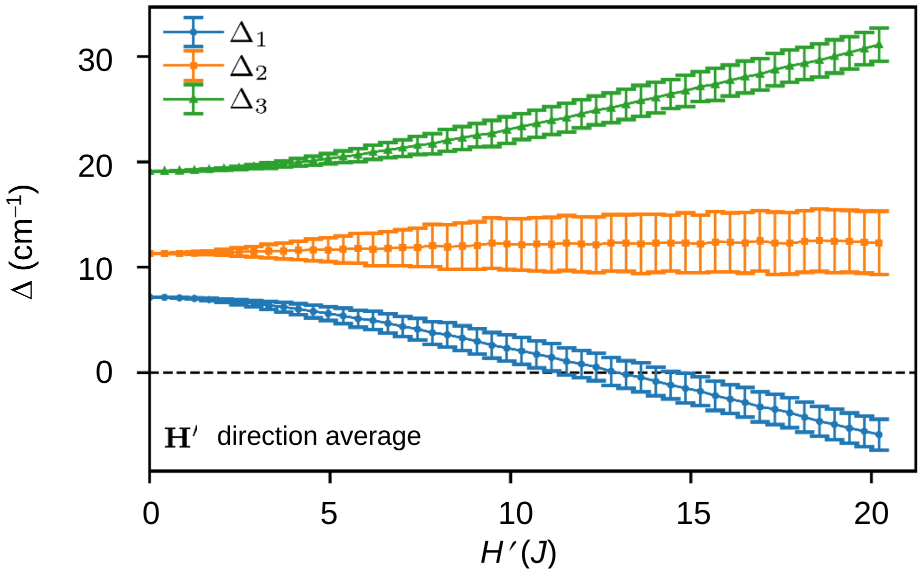

Supplementary Figure 16. Δ_1, Δ_2, Δ_3 as functions of the magnetic field strength averaged over all directions. The error bars denote the widths of the distributions, while the markers denote their means. Source data are provided as a Source Data file.

Both Δ_1 and Δ_2 contain mixings of the states $|1, \pm 1\rangle$. Due to the arrangement of the gaps, we expect Δ_1 to be the thermally more occupied relevant level, such that the signal seen in HFESR should mainly correspond to the energy difference $\Delta_3 - \Delta_1$. A comparison to the HFESR experimental data is given in Supplementary Figure 17. These results show a good agreement between theory and experiment.

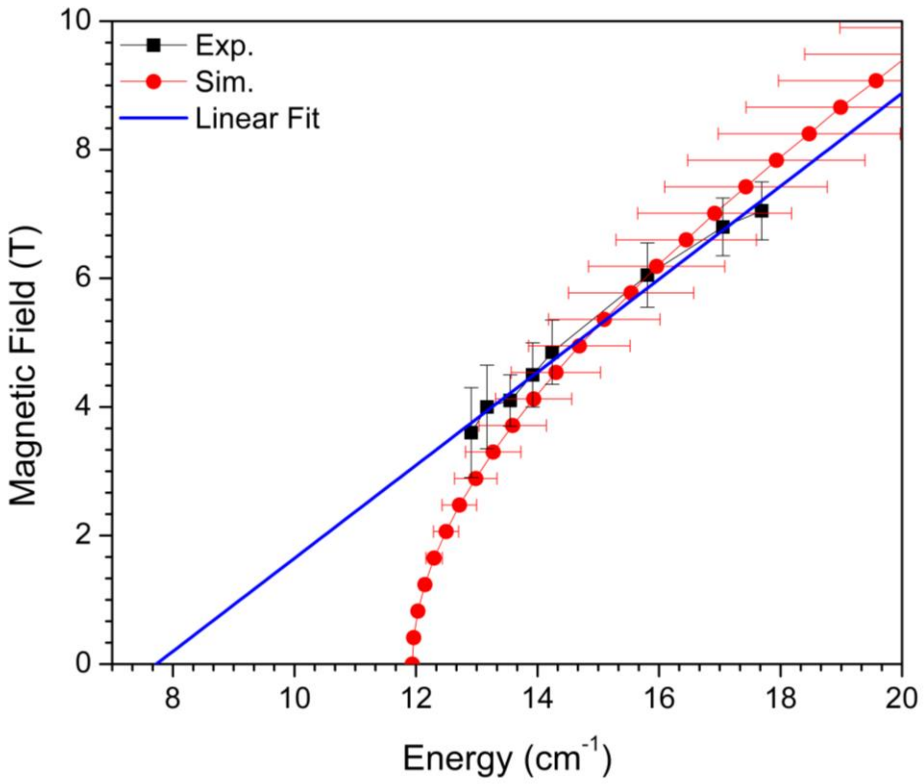

Supplementary Figure 17. Frequency dependence of the resonance of NiBO observed by HFESR and magnetic field strength vs. the difference $\Delta_3 - \Delta_1$ from the QMC simulations. The blue line is a linear fit to the data. In the experiment, error bars of the magnetic fields are shown. In the simulations, error bars in the energy $\Delta_3 - \Delta_1$ denote the width of the distribution. Source data are provided as a Source Data file.

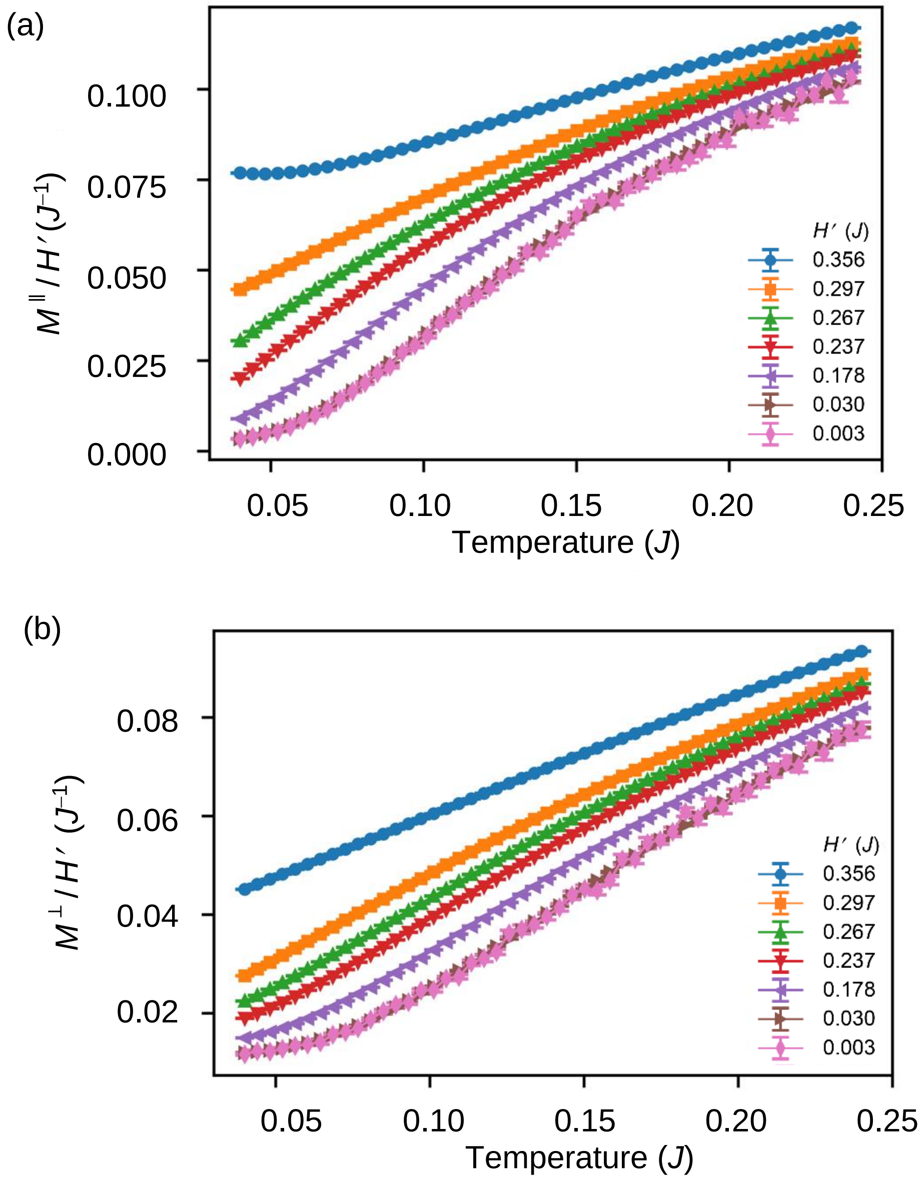

Supplementary Figure 18. (a) QMC results for the temperature-dependence of the magnetization divided by the field strength H' for finite magnetic fields parallel to the easy-axis direction. (b) QMC results for the temperature-dependence of the magnetization divided by the field strength H' for finite magnetic fields perpendicular to the easy-axis direction. Here, Boltzmann constant k_B, the Bohr magneton μ_B, and the Planck constant $\hbar$ are all set to 1. The magnetic field strength and temperature are then expressed in the unit of the exchange coupling constant J. Errors in $M^{\parallel}$ and $M^{\perp}$ are shown. Source data are provided as a Source Data file.

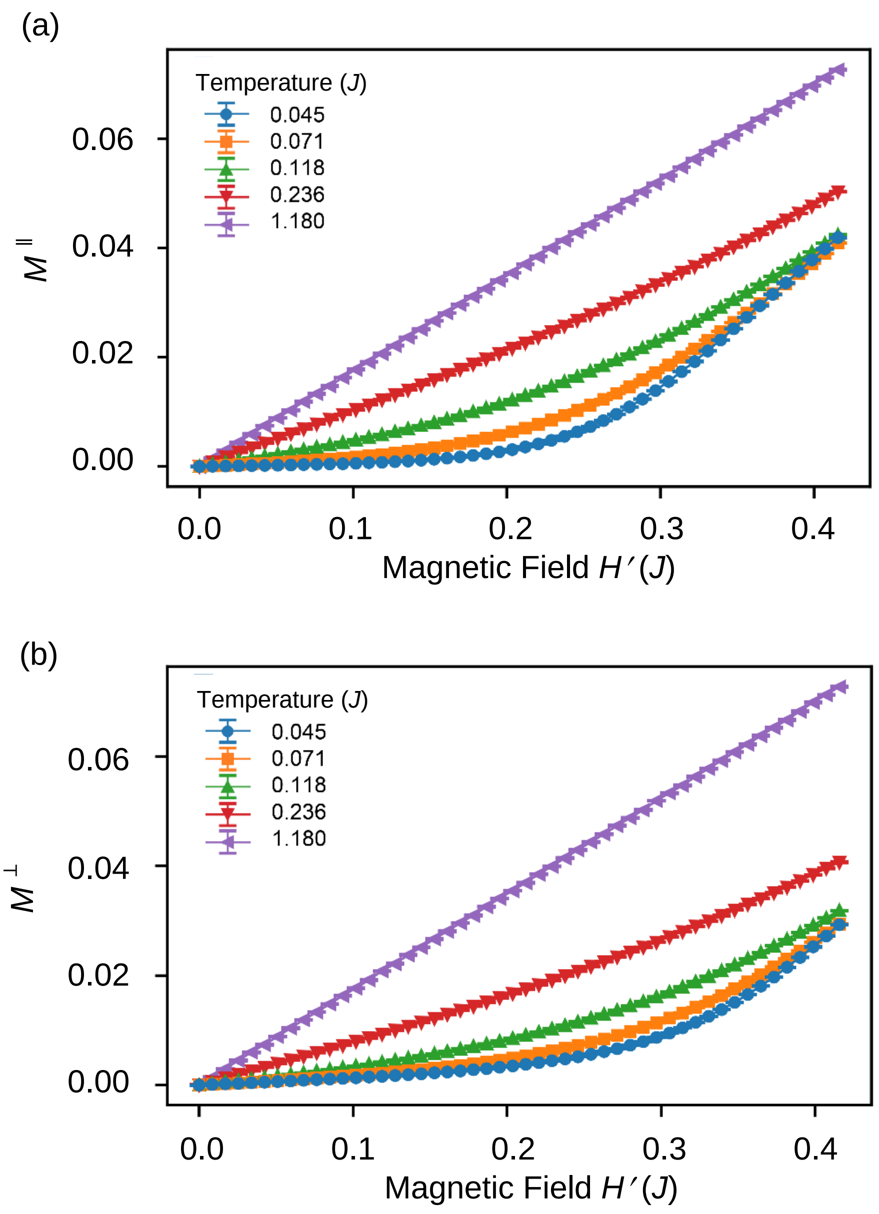

Supplementary Figure 19. (a) QMC results for the field-dependence of the magnetization in finite magnetic fields oriented parallel to the easy-axis direction at various temperatures. (b) QMC results for the field-dependence of the magnetization in finite magnetic fields oriented perpendicular to the easy-axis direction at various temperatures. See the caption of Supplementary Figure 18 for the unit in the figure. Errors in $M^{\parallel}$ and $M^{\perp}$ are shown. Source data are provided as a Source Data file.

Supplementary Note 6. *Additional specific-heat data*

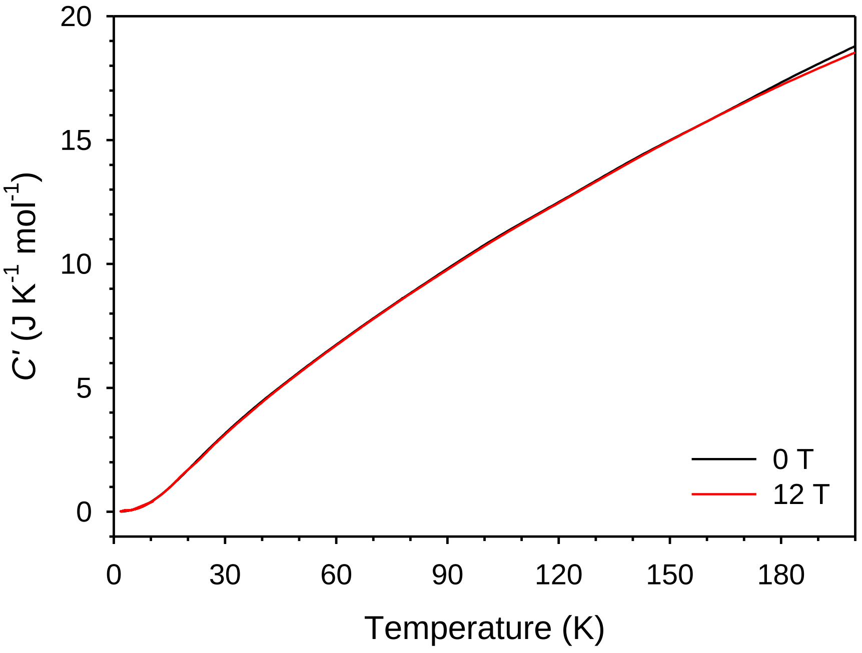

Supplementary Figure 20. Specific heat of NiBO at zero-field and 12 T between 1.92 K and 200 K. Source data are provided as a Source Data file.

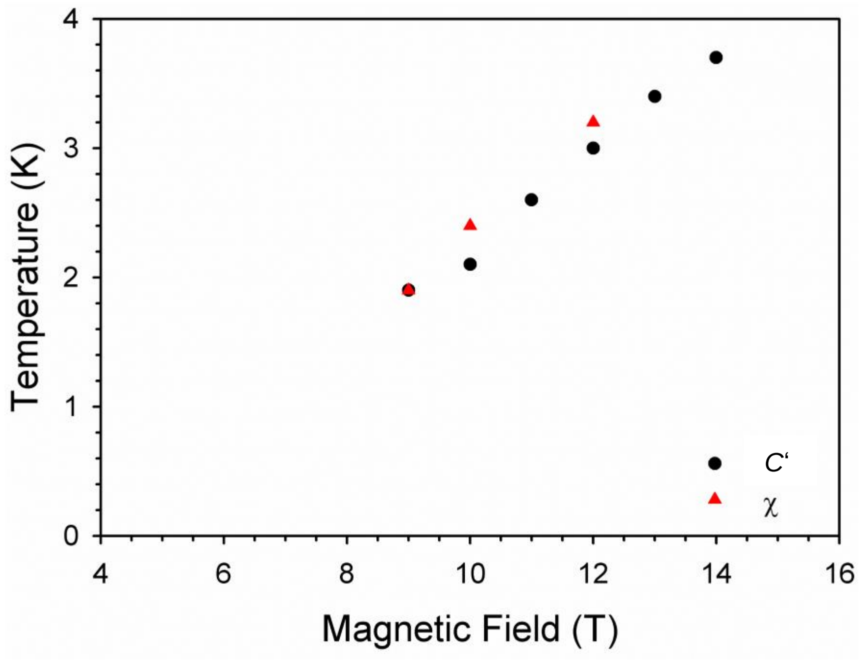

Supplementary Figure 21. Transition points estimated from specific heat and magnetization data of NiBO for magnetic field beyond 9 T. Source data are provided as a Source Data file.

Supplementary Note 7. *Additional inelastic neutron scattering (INS) spectra, tables of phonon symmetries, and spin densities*

Supplementary Figure 22 shows the comparison between the experimental INS spectrum of NiBO at 5 K and the calculated INS spectrum. The calculated spectrum compares reasonably well with the experimental data.

Supplementary Figure 23 gives a comparison of the INS spectrum of NiBO at 5 K with that of **NiBO-*d*$_{\mathbf{8}}$**. Deuteration with heavier D atoms in NiBO-d_8 shift many phonon peaks to lower energies. For example, the band around 3068 cm^{-1} in NiBO is most likely from C-H stretchings of the 4 4'-bipyridine ligand. The corresponding C-D stretching band in NiBO-d_8 appears at about 2307 cm^{-1}, a shift of 761 cm^{-1}.

Supplementary Tables 3 and 4 contain a list of phonon symmetries and modes for NiBO and NiBO-d_8, respectively, that are extracted from the DFT phonon calculations.

Comparison of the phonon features of NiBO between the 5 and 40 K is given in Supplementary Figure 24. At 40 K, the INS data fluctuate more in intensity than the 5 K data, due mostly to the thermal energy contribution. The data at both temperatures have similar phonon features.

In addition to the calculated phonon spectra, spin densities of NiBO were also obtained from the DFT calculations using VASP. Spin density provides insight into the localization of spin in the metal ions and the ligands in the system. The calculations do not include the spin densities in the bonds, but rather only show those in individual atoms inside a unit cell. The full list of spin densities is given in Supplementary Table 5. The results show that most of the spin densities are concentrated at the Ni^{2+} ion, containing 1.781 (89.1%) of the 2 unpaired electrons. The rest of the spin densities are dispersed among the ligands (oxalate and 4,4′-bipyridine). We

recently found that in the Co-TODA,[5] the spin densities concentrated in the Co^{2+} ions are 2.68-2.69 (89.3-89.7%) of the 3 unpaired electrons on the metal ions. The percentage of the spin concentration is comparable to the spin densities in the current Ni^{2+} system, which is possibly due to both compounds having only N and O coordinating atoms.

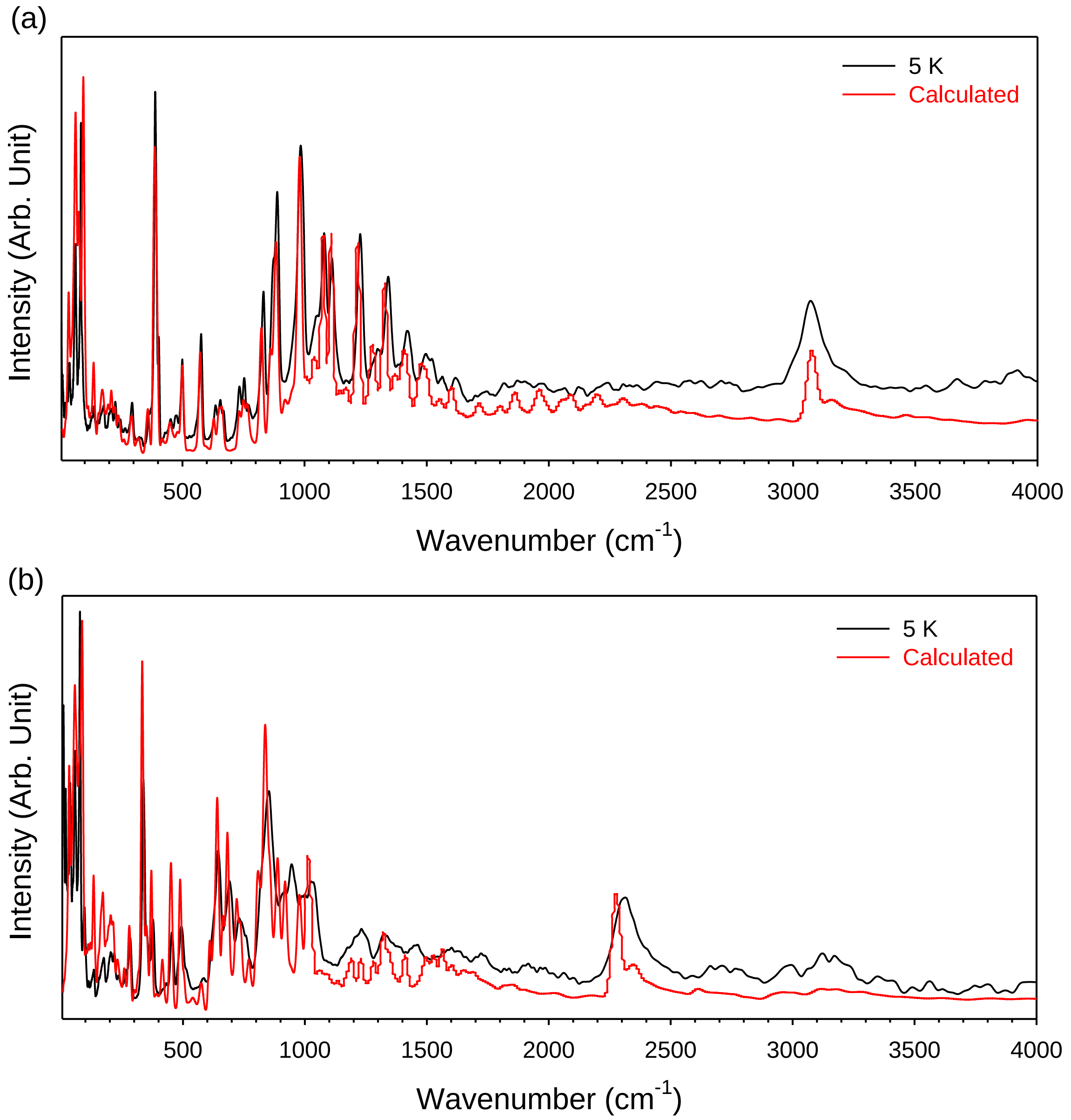

Supplementary Figure 22. Calculated phonon spectrum compared to the experimental INS spectrum at 5 K and 5-4000 cm^{-1}. (a) NiBO. (b) NiBO-d_8. Source data are provided as a Source Data file.

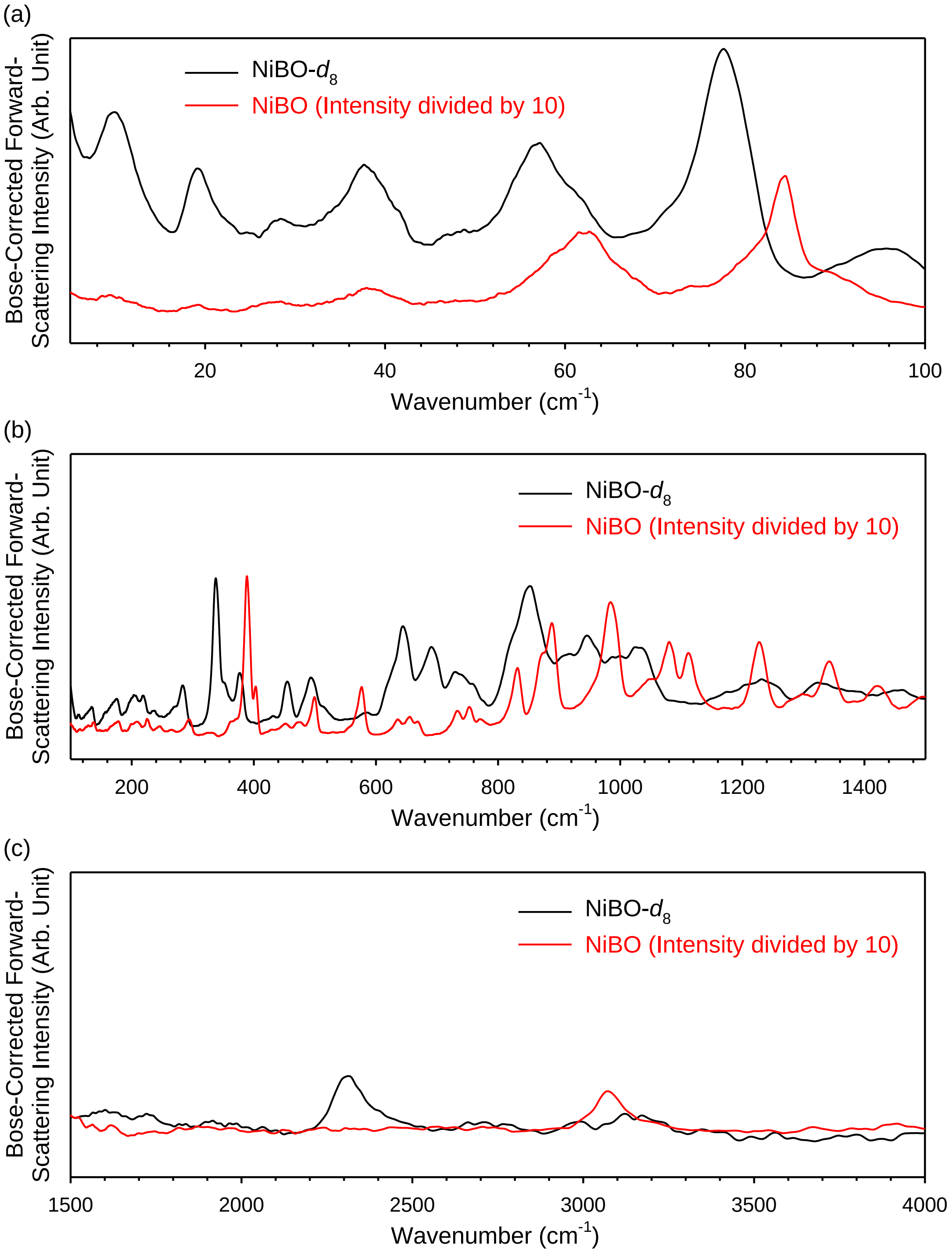

Supplementary Figure 23. Comparison of INS spectra of NiBO and NiBO-d_8 at 5 K. (a) 5-100 cm^{-1}. (b) 100-1500 cm^{-1}. (c) 1500-4000 cm^{-1}. Larger neutron scattering cross section of H atoms (82.0 barn) than D atoms (7.6 barn)[6] leads to more intense peaks in NiBO which are divided by 10 to compare with those of NiBO-d_8. Source data are provided as a Source Data file.

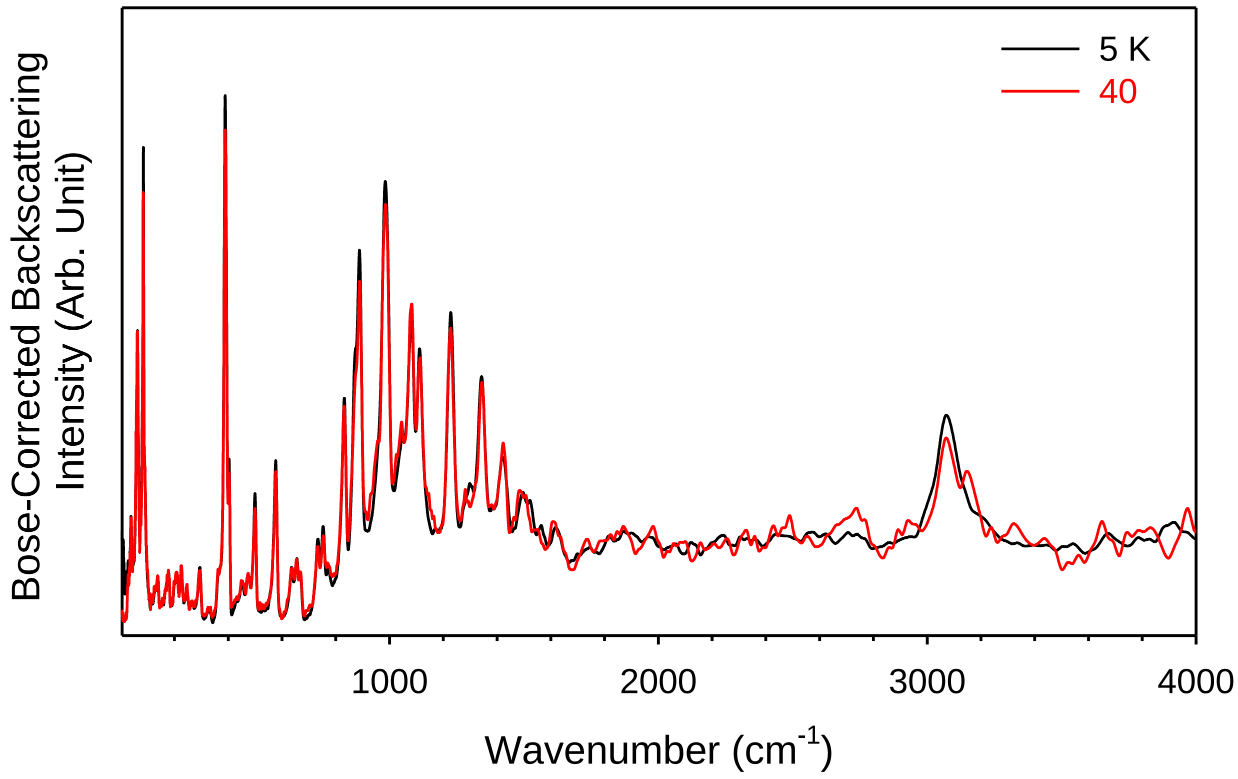

Supplementary Figure 24. INS spectra of NiBO at 5 and 40 K. The two spectra are similar; The latter has more vibrational intensity due to the increase in thermal energy compared to that at 5 K. Source data are provided as a Source Data file.

Supplementary Table 3. Calculated phonon symmetries and modes of NiBO

**Wavenumber (cm^{-1})**	**Symmetries of the modes**	**Wavenumber (cm^{-1})**	**Symmetries of the modes**
68.853	*B*	887.178	*A*
78.699	*B*	975.925	*B*
78.873	*A*	980.973	*A*
82.731	*A*	1002.163	*A*
108.143	*B*	1008.519	*A*
115.883	*B*	1033.115	*A*
182.689	*B*	1075.207	*A*
183.123	*A*	1077.428	*A*
192.065	*B*	1106.668	*B*
197.872	*B*	1108.590	*B*
211.306	*A*	1211.672	*A*
224.928	*B*	1223.726	*A*
244.372	*A*	1271.586	*B*
279.673	*A*	1284.012	*A*
282.652	*B*	1285.602	*B*
297.328	*B*	1313.346	*B*
318.915	*B*	1326.318	*B*
382.149	*A*	1331.750	*B*
391.352	*A*	1403.771	*B*
393.596	*B*	1405.542	*A*
415.794	*B*	1417.434	*B*
448.006	*A*	1475.766	*A*

498.541	*B*	1499.908	*A*
501.982	*A*	1523.854	*B*
569.796	*A*	1550.712	*B*
574.936	*B*	1588.764	*B*
628.673	*A*	1594.705	*A*
648.740	*B*	1596.594	*A*
664.476	*B*	1598.219	*A*
733.178	*B*	3068.039	*A*
749.623	*B*	3069.185	*B*
766.278	*A*	3070.673	*B*
773.847	*B*	3072.682	*A*
813.957	*B*	3079.726	*B*
817.366	*B*	3080.780	*B*
866.603	*B*	3081.382	*A*
868.397	*A*	3083.977	*A*
881.198	*A*		

Supplementary Table 4. Calculated phonon symmetries and modes of NiBO-d_8

**Wavenumber (cm^{-1})**	**Symmetries of the modes**	**Wavenumber (cm^{-1})**	**Symmetries of the modes**
67.764	*B*	806.778	*A*
72.247	*A*	814.405	*B*
75.419	*A*	818.540	*B*
76.935	*B*	831.743	*B*
106.011	*B*	837.226	*A*
113.001	*B*	838.394	*B*
179.715	*B*	844.040	*B*
181.083	*A*	857.073	*A*
185.443	*B*	875.488	*A*
194.502	*B*	886.364	*A*
211.253	*A*	919.773	*A*
217.190	*B*	974.144	*A*
243.463	*A*	983.786	*A*
279.658	*A*	1010.006	*A*
280.325	*B*	1011.138	*B*
285.463	*B*	1025.687	*B*
318.159	*B*	1191.678	*A*
330.272	*A*	1233.302	*B*
333.849	*A*	1278.371	*B*
346.585	*A*	1313.183	*B*
368.566	*B*	1322.399	*B*
414.149	*B*	1324.163	*B*

447.946	*A*	1344.863	*A*
448.834	*B*	1405.512	*A*
492.116	*B*	1414.194	*A*
501.797	*A*	1496.736	*B*
569.768	*A*	1529.450	*B*
610.661	*A*	1562.073	*A*
625.308	*B*	1563.919	*A*
638.532	*B*	1588.112	*B*
640.151	*B*	1594.830	*A*
644.741	*B*	2265.047	*B*
661.914	*B*	2265.357	*B*
678.488	*A*	2268.906	*A*
686.089	*A*	2271.543	*A*
721.909	*A*	2280.762	*B*
737.447	*B*	2280.899	*B*
774.349	*B*	2285.049	*A*
803.314	*A*	2287.734	*A*

Supplementary Table 5. Calculated spin densities of atoms in NiBO [one Ni(μ-4,4′-bpy)(μ-ox) unit]

**Atom**	**Spin Density**
Ni atom	1.781
All O atoms	0.035
Both N atoms	0.035
C (in oxalate)	–0.001
C (*ortho* from the N atoms in bpy)	–0.001
C (*para* from the N atoms in bpy, i.e., C atom linked to the other ring)	–0.001
C (*meta* from the N atoms in bpy)	0.002
All H atoms	<0.001
**Total**	**1.995**

Supplementary References

1 Lu, J. Y., Lawandy, M. A., Li, J., Yuen, T. & Lin, C. L. A New Type of Two-Dimensional Metal Coordination Systems: Hydrothermal Synthesis and Properties of the First Oxalate−bpy Mixed-Ligand Framework [M(ox)(bpy)] (M = Fe(II), Co(II), Ni(II), Zn(II); ox = $C_2O_4^{2-}$; bpy = 4,4′-bipyridine). *Inorg. Chem.* ***38***, 2695-2704 (1999).

2 Mugiraneza, S. & Hallas, A. M. Tutorial: a beginner's guide to interpreting magnetic susceptibility data with the Curie-Weiss law. *Commun. Phys.* **5**, 95 (2022).

3 Golinelli, O., Jolicoeur, T. & Lacaze, R. The magnetic field behaviour of a Haldane-gap antiferromagnet. *J. Phys.: Condens. Matter* **5**, 7847 (1993).

4 Golinelli, O., Jolicoeur, T. & Lacaze, R. Haldane gaps in a spin-1 Heisenberg chain with easy-plane single-ion anisotropy. *Phys. Rev. B* **45**, 9798-9805 (1992).

5 Tin, P. *et al.* Advanced Spectroscopic and Computational Studies of a Cobalt(II) Coordination Polymer with Single-Ion-Magnet Properties. *J. Phys. Chem. C* **126**, 13268-13283 (2022).

6 Neutron scattering lengths and cross sections of the elements and their isotopes. *Neutron News* **3 (Issue 3)**, 29-37 (1992).
